# Supplementary material for: Post-Electric Current Treatment Approaching High-Performance Flexible n-Type Bi2Te3 Thin Films
Source: Micromachines (Basel). 2022 Sep 17;13(9):1544. doi: 10.3390/mi13091544 (PMC9505272; doi:10.3390/mi13091544)
Supplement: Supplementary file 1 [file micromachines-13-01544-s001.zip › micromachines-1920953-supplementary.pdf]

Post-electric current treatment approaching high-performance flexible n-type Bi_2Te_3 thin films

Dongwei Ao [1, #,*], Wei-Di Liu [2, #], Fan Ma [3], Wenke Bao [1], Yuexing Chen [4]

Figure S1 Calculated crystallinity of 0.5 A - Bi_2Te_3 f-TF.

Figure S2 (a) The SEM images of 0.6 A - Bi_2Te_3 thin film; (R-a) The repetitive SEM images of the 0.6 A - Bi_2Te_3 thin film.

Figure S3 The repetitive test of TE performance of the 0.5 A - Bi_2Te_3 thin film.

Figure S4 (a,b) The repetitive test result of the bending resistance of 0.5 A - Bi_2Te_3 thin film at bending radius of 9 mm and bending cycle of 800, respectively.

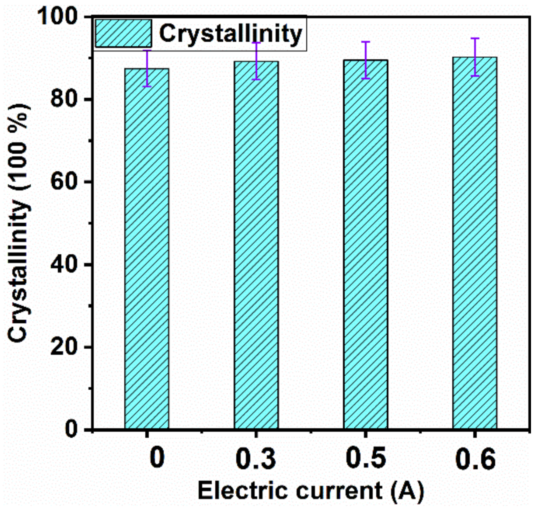

Figure S1. Calculated crystallinity of 0.5 A - Bi_2Te_3 f-TF.

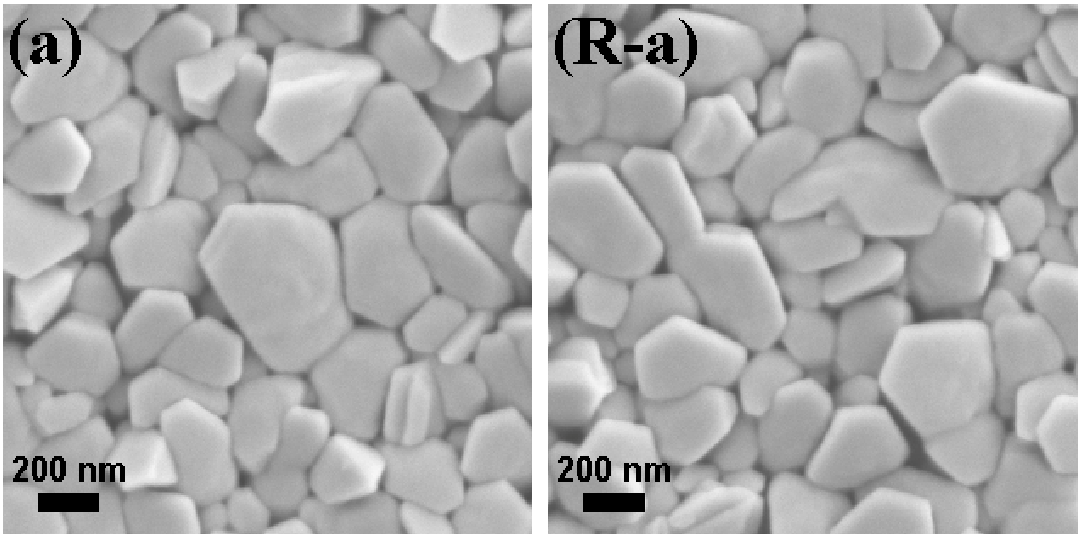

Figure S2. (a) The SEM images of 0.6 A - Bi_2Te_3 thin film. (R-a) The repetitive SEM images of the 0.6 A - Bi_2Te_3 thin film.

The repetitive SEM image was provided as shown in Figure R3. After 3 measure cycles and keep for a long time, the repetitive SEM images (**Figure S**1b) of the 0.6 A - Bi_2Te_3 thin film is basically the same with the **Figure S**1a.

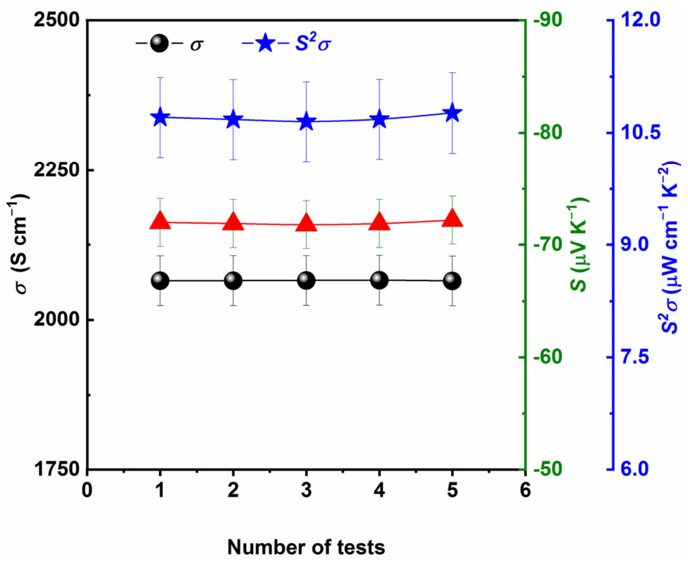

Figure S3. The repetitive test of TE performance of the 0.5 A - Bi_2Te_3 thin film.

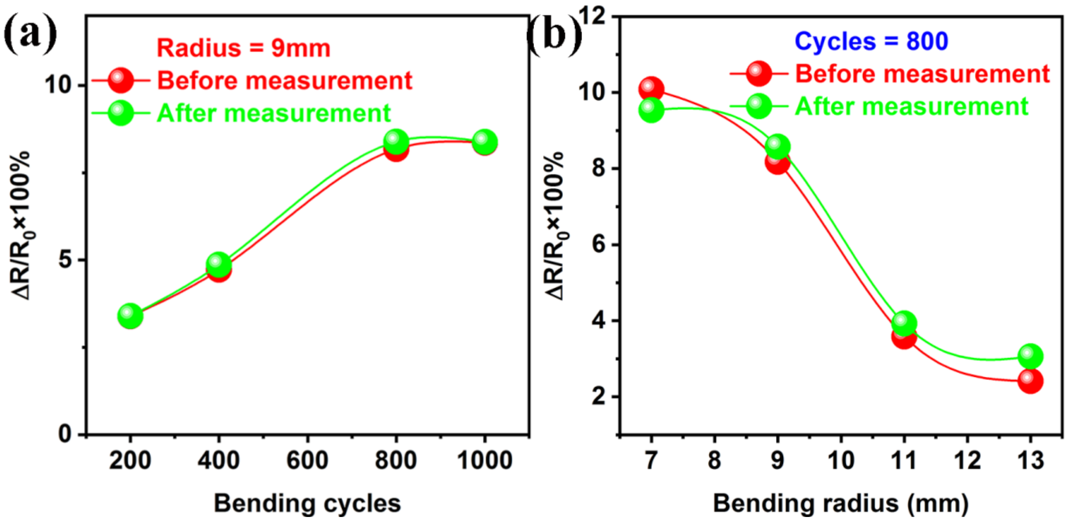

Figure S4. (a,b) The repetitive test result of the bending resistance of 0.5 A - Bi_2Te_3 thin film at bending radius of 9 mm and bending cycle of 800, respectively.

In this work, the n_e of the thin films ranges from 2.03×10^{20} to 3.84×10^{20} cm^{-3}, which is higher than that of bulk Bi_2Te_3 ($1\sim9 \times 10^{19}$ cm^{-3}) [1-3], which is responsible for the low Seebeck coefficient of the thin films.

1. Zhu, B.; Liu, X.; Wang, Q.; Qiu, Y.; Shu, Z.; Guo, Z.; Tong, Y.; Cui, J.; Gu, M.; He, J. Q. Realizing record high performance in n-type Bi_2Te_3-based thermoelectric materials. Energy Environ. Sci., 2020, 13, 2106.

2. Hao, F.; Xing,T.; Qiu, P.; Hu, P.; Wei, T.; Ren, D.; Shi, X.; Chen, L.; Enhanced Thermoelectric Performance in n-Type Bi_2Te_3-Based Alloys via Suppressing Intrinsic Excitation. ACS Appl. Mater. Interfaces 2018, 10, 21372−21380.

3. Shi, X. L.; Zou, J.; Chen, Z. G. Advanced Thermoelectric Design: From Materials and Structures to Devices. *Chem. Rev.* **2020**, 120, 7399-7515.
